# Isolated Dysplasia Epiphysealis Hemimelica (Trevor Disease) of the Acetabulum: Literature Review

**DOI:** 10.5435/JAAOSGlobal-D-23-00138

**Published:** 2023-10-06

**Authors:** Gregory Benes, Alexandra Seidenstein, Aaron Brandt

**Affiliations:** From the Department of Orthopaedic Surgery, The Johns Hopkins University, Baltimore, MD.

## Abstract

Dysplasia epiphysealis hemimelica (DEH), also known as Trevor disease, is a rare pathologic proliferation of cartilage with unknown etiology creating cartilaginous osteochondroma exostoses intra-articularly or juxta-articularly. Herein, we reviewed the literature about acetabular osteochondroma in children and report a case of a 9-year-old boy who presented to the orthopaedic clinic with complaints of gait disturbance, right hip discomfort, and with increasing severity and frequency of hip subluxation episodes over the course of a year. Imaging studies revealed dysplasia of the right hip with subluxation secondary to acetabular lesion. The patient underwent surgical hip dislocation to facilitate surgical excision of the lesion and reduce hip, and pathology confirmed osteochondroma with chondromatosis. We report the early follow-up for this patient and discuss the value of surgical hip dislocation to manage intra-articular bone or cartilage lesions.

Dysplasia epiphysealis hemimelica (DEH) (or Trevor disease) is a rare pathologic proliferation of cartilage with unknown etiology. The condition creates cartilaginous exostoses inside a joint or juxta-articularly.^1^ The evolving epiphyseal lesions are identical to osteochondromas histologically.^1^ When they originate from articular cartilage, the lesions are able to project into the joint cavity and characteristically only involve half of the joint, hence its name hemimelica.^1,2^

Usually, children and young adults are afflicted, with men more frequently than women, in a ratio of 3:1.^2,3^ The most common locations are the epiphyses of the lower limb with a predisposition for the medial femoral condyle, the distal aspect of the tibia, and the talus.^1,2^ Typical patient presentation includes painless swelling or asymmetric gait because of localized overgrowth of cartilage.^2,4^ Intra-articular acetabular osteochondroma have been reported in cases of multiple hereditary exostoses; however, isolated intra-articular acetabular osteochondroma, as seen in our patient, have only been reported in a small number of previous cases.^2,3,5-7^

Herein, we report a 9-year-old boy with Trevor disease involving the hip causing hip subluxation, instability, and gait disturbances. In addition to providing a concise, contemporary review of the literature, our report is the first to include detailed intraoperative photography. The patient and family agreed to the submission and publication of this case.

## Case Report

### Clinical Presentation

A 9-year-old boy presented to the orthopaedic clinic for evaluation of right hip discomfort with instability and limp. He began having right hip subluxation events 1 year ago, which he described as hip painless hip “popping” events. Over the 2 months before presentation, events progressively worsened in severity and increased frequency to 2 to 3 times per week. He remained active; however, the hip subluxation occurred even with minimal activity such as rolling over in bed. Most recent episode occurred while playing flag football where the hip reportedly could not be reduced requiring presentation to the emergency department. No formal reduction was required as his hip “popped” and felt better, but radiographs were obtained for the first time.

### Physical Examination

The patient was noted to have a mild Trendelenburg gait and sign but denied pain. Hip range of motion was limited and notable apprehension with external rotation (sensation of hip popping out at 40°) and abduction (lacked 15° compared with contralateral hip). Not previously noted by family, but his right greater trochanter was much more prominent than the left.

### Imaging Studies

**Figure 1:**
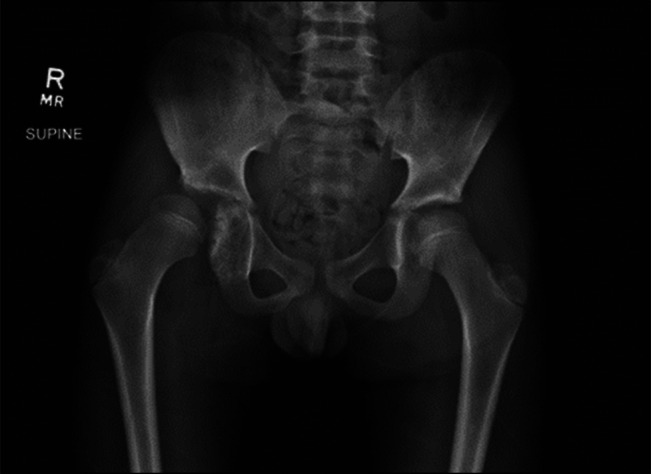
Initial radiograph showing mass lesion in right acetabulum causing subluxation of the right hip and abnormal development of right hip with elevated acetabular index and blunting of lateral sourcil.

**Figure 2:**
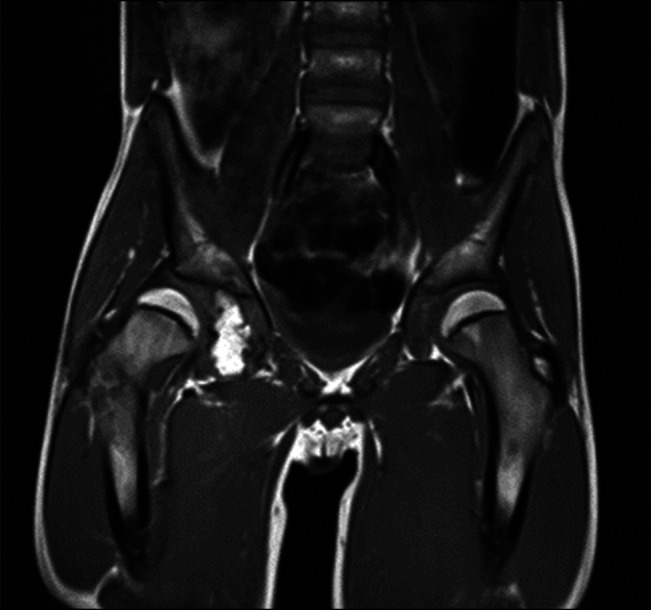
MRI of the pelvis showing osseus abnormality involving the base of the right acetabulum and extending to the triradiate approximately 5.5 × 2.5 cm confluent with base of acetabulum. Minimal cartilage cap.

**Figure 3:**
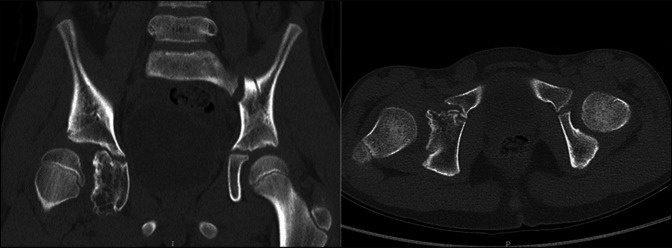
CT imaging demonstrating bony abnormality of inferior acetabulum

**Figure 4:**
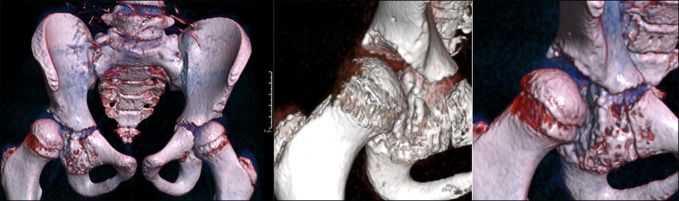
Three-dimensional reconstructed CT imaging showing intra-articular acetabular lesion

Imaging, including radiograph (Figure 1), MRI (Figure 2), and CT (Figures 3 and 4) scans, demonstrated a large osteochondral lesion originating from the fovea of the acetabulum.

### Surgical Intervention

**Figure 5:**
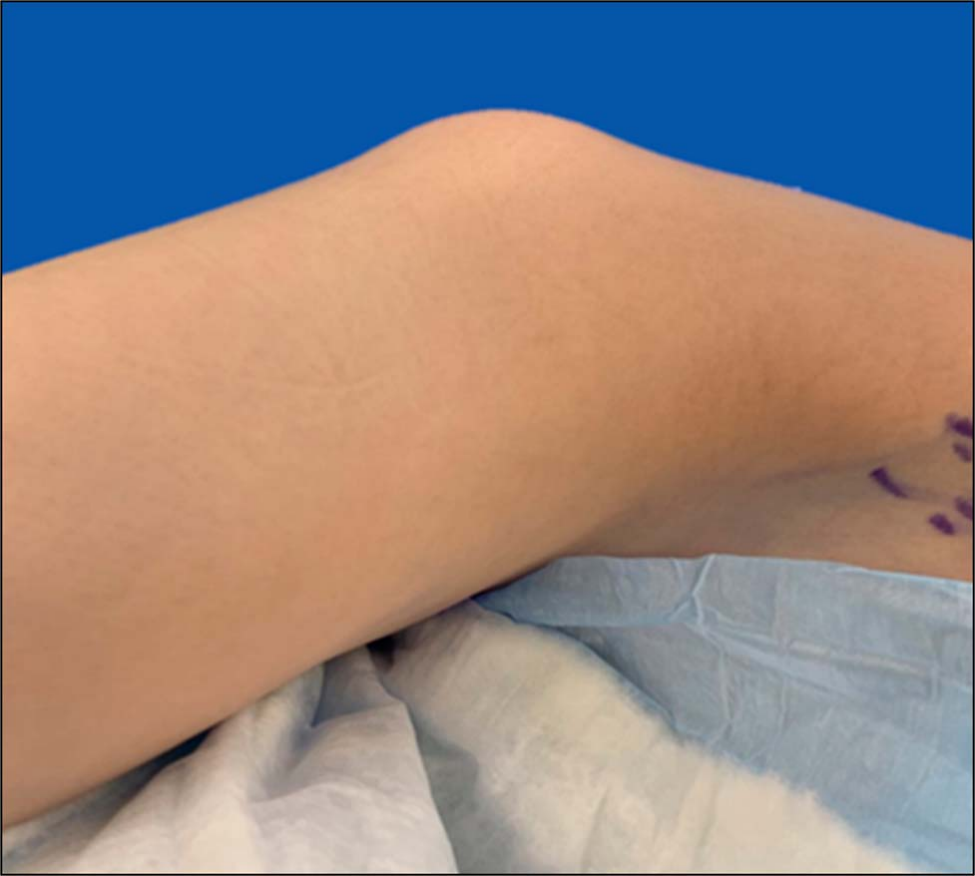
Photograph showing preoperative positioning of the patient in a lateral decubitus position with lateral right hip prominence

Lateral right hip prominence was apparent on preoperative lateral decubitus position (Figure 5). We approached the hip using a modified Gibson approach and trochanteric flip described by Ganz.^8,9^ The dissection and trochanteric osteotomy were performed in the standard fashion. After performing capsulotomy, multiple osteochondral loose bodies were pushed out of the hip joint and removed. The hip was notably stiff, and while we were able to observe the hip subluxating with minimal rotation, visualization of the hip was still challenging given the size of the lesion and tension on the capsule. We were able to palpate the lesion which was molded well to the femoral head and acting as a false acetabulum.

**Figure 6:**
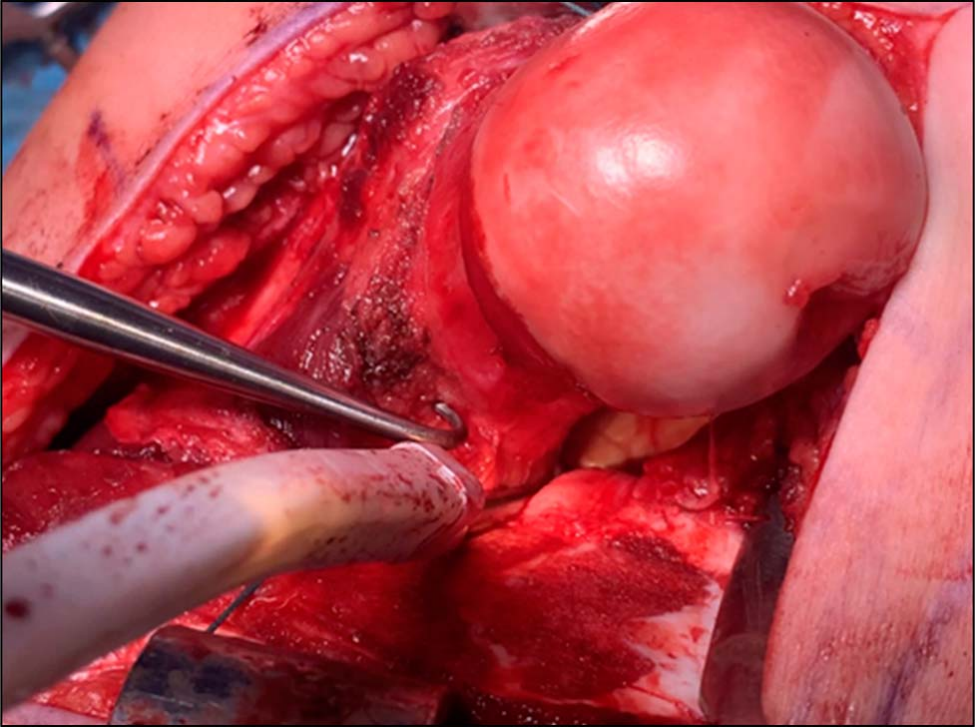
Intraoperative photograph showing healthy appearance of femoral head without deformity. Acetabular lesion notable with osteochondral loose body.

**Figure 7:**
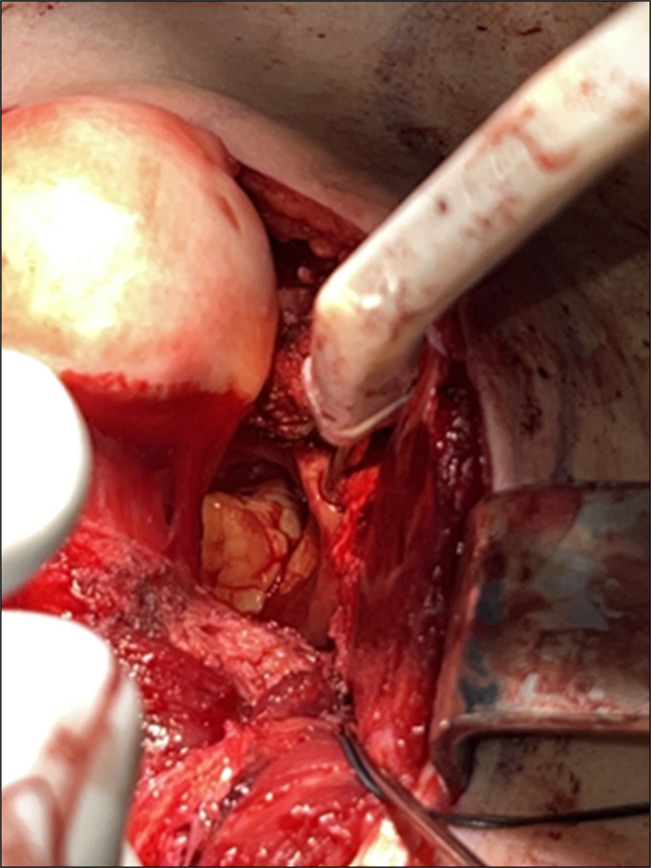
Image showing visualization of intra-articular osteochondroma

**Figure 8:**
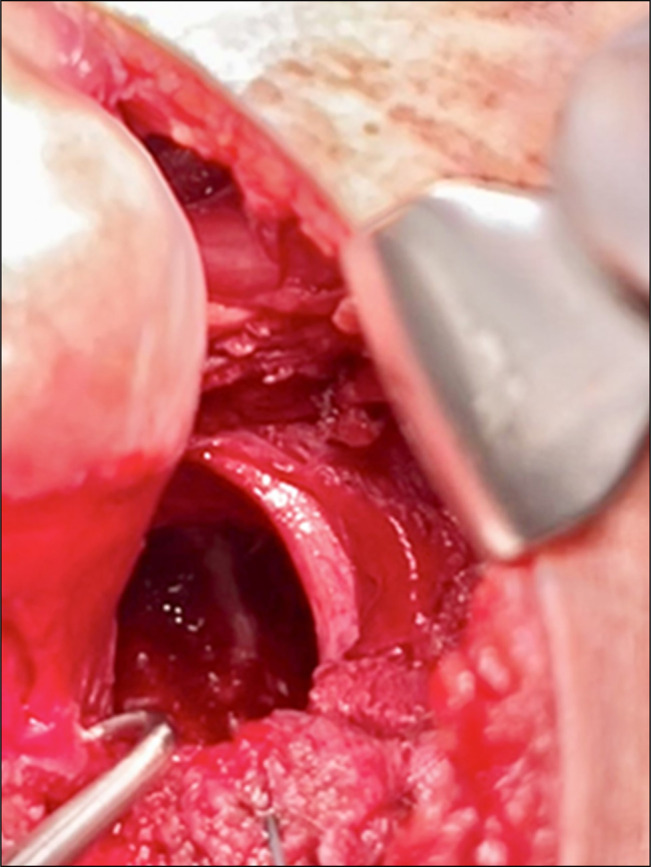
Image showing visualization of acetabulum after lesion excision and acetabuloplasty

**Figure 9:**
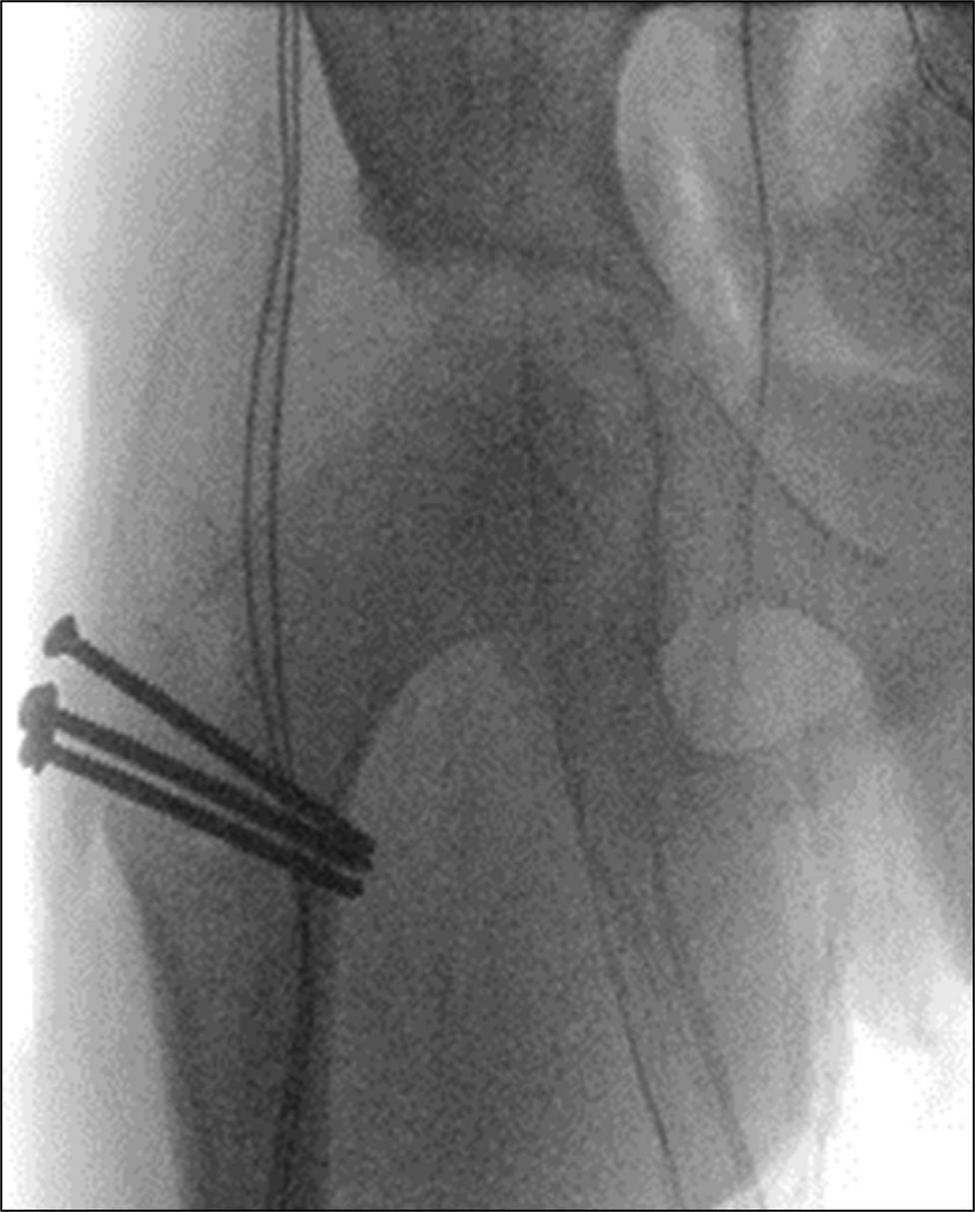
Intraoperative fluoroscopic images confirming that hip was reduced.

The hip was slowly and carefully dislocated to visualize the acetabulum. The labrum was visualized circumferentially from the 10 to 3 o'clock position with no visible injury or abnormality (Figure 6). The lesion (Figure 7) obstructed the view of the peripheral portion of the acetabulum, and we determined that to safely dislocate the hip without tension, we needed to excise a portion of the lesion. We used a curved gauge to resect the main lesion. To try to protect the triradiate cartilage which appeared to be preserved under the lesion, we removed the lesion in a piecemeal fashion and under direct visualization (Figure 8). We then were able to evaluate the rest of the acetabulum. The femoral head was then reduced to evaluate the position, but there was persistent evidence of subluxation; therefore, a more aggressive acetabuloplasty was performed at the fovea. Most of the acetabular cartilage had an irregular appearance, which was similar to the surface of the lesion, but was congruent with the femoral head. The main lesion appeared to be from the fovea and anterior acetabulum, so the superior/posterior acetabuloplasty was limited to preserve the cartilage. While he did have some residual dysplasia, the head reduced well and had good coverage (Figure 9), so we elected to not perform acetabular osteotomy and monitor closely. We performed capsulorrhaphy to remove redundant capsular tissue and help stabilize the hip.

### Postoperative Course

**Figure 10:**
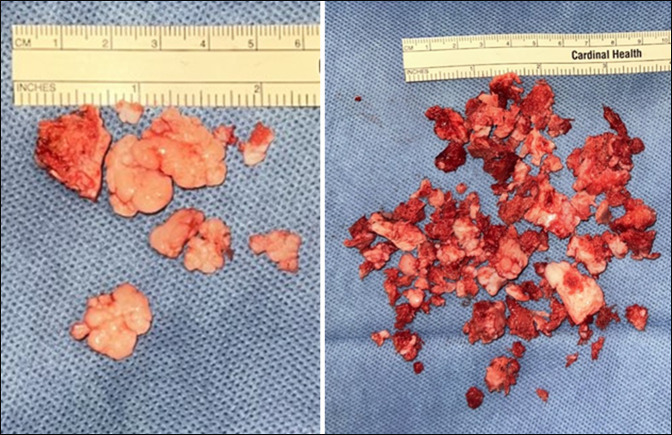
Images showing gross specimens including loose intraarticular pieces (left, measuring 4.0 × 3.8 × 1.0 cm in aggregate) and resected osteochondroma with cartilaginous cap of variable thickness (right, measuring 8.0 × 6.5 × 1.4 cm in aggregate)

**Figure 11:**
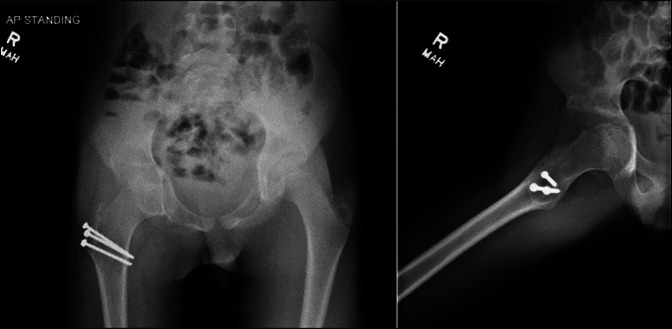
Radiographs at 4 months postoperatively showing right femoral head seated in acetabulum with medialization of hip center, acetabular protrusio relative to contralateral side. No evidence of recurrence or subluxation.

Surgical pathology of the specimens was diagnosed as osteochondromas, and loose fragments were consistent with chondromatosis (Figure 10). Postoperatively, the patient was maintained in a hip abduction brace when out of bed and while sleeping for 3 weeks. He was kept toe touch weight bearing with use of crutches for 6 weeks. At 4 months postoperatively, he had noted improvement in strength and mobility. He continues to work with physical therapy to address ongoing stiffness which may be related to capsulorrhaphy or postoperative brace, but he denies any instability events since surgery. He was able to achieve more than 90° of hip flexion with slightly limited internal (lacking 15°) and external (lacking 35°) rotation. No evidence of leg length discrepancy was noted. He has returned to sports (flag football and rollerblading) with no pain. Imaging showed residual dysplasia and medialization of hip center relative to normal side but no subluxation or recurrence in this early period (Figure 11).

## Discussion

DEH was first described in 1926 by the French surgeons Mouchet and Belot as “tarsomegalie” to describe an intraarticular lesion of a tarsal bone.^10^ Further reports of eight pediatric patients by Trevor described a more widespread lesion that involved the talus, malleoli, the navicular, and all the cuneiform bones.^11^ In the first-known English report, Trevor believed the lesion was congenital, and the etiology was related to an insult during the formation of the limb bud.^10,11^ Six years later, Fairbank reported on 14 patients and officially named the condition DEH as he believed the process to be “a true dysplasia or faulty growth of part of the epiphysis itself.^12^”

DEH lesions typically enlarge during growth years and are generally treated surgically because of their location, which makes understanding the natural progression of the affected joint challenging.^1^ The mechanical effect of an enlarging mass restricts joint motion while the stimulating effect of these lesions can either accelerate or, less commonly, decrease limb growth, potentially resulting in limb length discrepancies.^1^ Therefore, surgery at an early stage is widely endorsed to better restore the normal growth, prevent the secondary degenerative arthritis, and re-establish motion of the afflicted joint.^3,5^ Linke et al^3^ reported a case of isolated DEH of the triradiate acetabulum cartilage, managed with surgical hip dislocation and mass excision, demonstrated secondary dysplasia 22 months postoperatively. While the suggested recurrence may be due to a local bone reaction of the disease leading to a secondary dysplasia of the afflicted hip, Linke et al^3^ concluded that removal of mass at earlier stages is imperative.

Few previous cases of isolated acetabular osteochondroma have been reported.^2,3,6,7^ Bleshman and Levy^7^ described an isolated osteochondroma of the acetabulum who reported discomfort and limp treated with surgical excision. Skaggs reported two cases of intra-articular osteochondroma of the acetabulum, where both lesions were removed through an anterior approach with femoral head dislocations. One of the patients had a recurrence of the lesion at 3 years after the index operation leading to another excision. There was excellent remodeling of the acetabulum at both 3 and 8 years after the operation, and the joint space was minimally narrowed.^2^ Wenger and Adamcyzk described a case of a 7-year-old girl with an extensive intra-articular lesion involving the superior and posterior articular surface of the acetabulum. In addition to a similar anterior approach with hip dislocation, the patient underwent triple innominate osteotomy plus femoral osteotomy to ensure hip stability with no evidence of recurrence at 3-year follow-up.^6^ No documented cases managed with temporary intraoperative dislocation of the hip have reported signs of long-term sequelae, such as osteonecrosis of the femoral head.^2,3,6^

With the symptoms and subluxation seen in our patient, we felt similarly that surgical excision with primary goal of reduction of the hip joint was the optimal treatment. We felt that surgical dislocation allowed for the best visualization of the lesion to allow excision and reduce risk of damage to triradiate cartilage and surrounding cartilage.^2,3,5,6^ Acetabular osteochondromas have been resected without femoral head dislocation in some cases; however, postoperative follow-up periods in these instances were limited to 3 and 14 months.^13^ Recurrence, as demonstrated in one of the cases described by Skaggs et al,^2^ remains a possibility within that timeframe. This report and review are presented to add to the literature on this rare pathology, and we will monitor this patient closely for recurrence, remodeling, and long term outcomes.
